# LASSO and Bioinformatics Analysis in the Identification of Key Genes for Prognostic Genes of Gynecologic Cancer

**DOI:** 10.3390/jpm11111177

**Published:** 2021-11-11

**Authors:** Shao-Hua Yu, Jia-Hua Cai, De-Lun Chen, Szu-Han Liao, Yi-Zhen Lin, Yu-Ting Chung, Jeffrey J. P. Tsai, Charles C. N. Wang

**Affiliations:** 1 School of Medicine, College of Medicine, China Medical University, Taichung 404333, Taiwan; jimura81@gmail.com; 2 Department of Emergency Medicine, China Medical University Hospital, Taichung 404333, Taiwan; 3 Institute of Statistical Science, Academia Sinica, Taipei 11529, Taiwan; as6309123@yahoo.com.tw; 4 Department of Bioinformatics and Medical Engineering, Asia University, Taichung 41354, Taiwan; delunchen.0509@gmail.com (D.-L.C.); liaoszuhan56@gmail.com (S.-H.L.); yizhenlin@asia.edu.tw (Y.-Z.L.); jjptsai@gmail.com (J.J.P.T.); 5 Department of Emergency Medicine, Asia University Hospital, Taichung 413505, Taiwan; ercyt0502@gmail.com; 6 Center for Precision Medicine Research, Asia University, Taichung 41354, Taiwan

**Keywords:** cervical cancer, endometrial cancer, bioinformatics, LASSO regression, prognostic biomarkers

## Abstract

The aim of this study is to identify potential biomarkers for early diagnosis of gynecologic cancer in order to improve survival. Cervical cancer (CC) and endometrial cancer (EC) are the most common malignant tumors of gynecologic cancer among women in the world. As the underlying molecular mechanisms in both cervical and endometrial cancer remain unclear, a comprehensive and systematic bioinformatics analysis is required. In our study, gene expression profiles of GSE9750, GES7803, GES63514, GES17025, GES115810, and GES36389 downloaded from Gene Expression Omnibus (GEO) were utilized to analyze differential gene expression between cancer and normal tissues. A total of 78 differentially expressed genes (DEGs) common to CC and EC were identified to perform the functional enrichment analyses, including gene ontology and pathway analysis. KEGG pathway analysis of 78 DEGs indicated that three main types of pathway participate in the mechanism of gynecologic cancer such as drug metabolism, signal transduction, and tumorigenesis and development. Furthermore, 20 diagnostic signatures were confirmed using the least absolute shrink and selection operator (LASSO) regression with 10-fold cross validation. Finally, we used the GEPIA2 online tool to verify the expression of 20 genes selected by the LASSO regression model. Among them, the expression of PAMR1 and SLC24A3 in tumor tissues was downregulated significantly compared to the normal tissue, and found to be statistically significant in survival rates between the CC and EC of patients (*p* < 0.05). The two genes have their function: (1.) PAMR1 is a tumor suppressor gene, and many studies have proven that overexpression of the gene markedly suppresses cell growth, especially in breast cancer and polycystic ovary syndrome; (2.) SLC24A3 is a sodium–calcium regulator of cells, and high SLC24A3 levels are associated with poor prognosis. In our study, the gene signatures can be used to predict CC and EC prognosis, which could provide novel clinical evidence to serve as a potential biomarker for future diagnosis and treatment.

## 1. Introduction

Gynecologic cancer is a type of malignant tumor that begins in the female reproductive system. Of all the gynecologic cancers, cervical cancer (CC) and endometrial cancer (EC) are the most common tumors of the female genital tract in the world, followed by ovarian cancer [1]. In recent years, numerous studies have demonstrated that abnormally expressed tumor markers may be involved in cancer initiation and progression, such as p16INKa/ki-67, E6/E7, PTEN, and ANXA2 [2,3,4,5,6]. Despite large efforts to develop novel biomarkers, cervical and endometrial cancers continue to be a serious health problem among women [7,8]. Patients with early stage (or locally advanced) CC and EC have access to a standard treatment comprising a combination of surgery, radiotherapy, and chemotherapy [9,10,11]. However, precise biomarkers and targeted therapy for CC and EC remain limited [12,13,14].

With the development of gene chip technology, understanding cervical and endometrial cancers from the perspective of the genome and proposing more effective biomarker genes provide potentially relevant information for clinical drug development [15]. Although CC and EC are two completely different types of cancer with different courses, symptoms, and treatments, it has been hypothesized that certain tumor-specific markers and a shared molecular mechanism in both cancers may be common to their tumorigenesis and development [16,17,18]. Therefore, a comprehensive analysis is required to improve understanding of these two types of tumor.

One of the popular machine learning models is the LASSO regression, which is a high-dimensional gene expression data analysis method that performs both feature selection and classification [19,20]. In our study, we analyzed gene expression profiles of CC and EC patients from the GEO public database in order to understand early molecular changes as well as biological mechanisms. For the early diagnosis of patients, we established a LASSO regression model to develop a gene signature for predicting gynecologic cancer. Analysis of overall survival (OS) in the cohort of cervical squamous cell carcinoma and endocervical adenocarcinoma (CESC) and uterine corpus endometrial carcinoma (UCEC) downloaded from TCGA allowed us to identify potential biomarkers of gynecologic cancer. The goal of our study was to identify early diagnostic molecules and improve the survival of patients.

## 2. Materials and Methods

### 2.1. Microarray Data Mining in Gene Expression Omnibus (GEO)

Raw microarray data of CC (GSE9750, GSE7803, GSE63514) and EC (GSE17025, GSE115810, GSE36389) were downloaded from the GEO database (Table 1). In the six datasets of our study, GSE9750, GSE7803, GSE115810, and GSE36389 were processed using the GPL96 platform (Affymetrix Human Genome U133A Array, HG-U133A, https://www.ncbi.nlm.nih.gov/geo/query/acc.cgi?acc=GPL96, accessed on 3 July 2021), while GSE63514 and GSE17025 were based on GPL570 platform (Affymetrix Human Genome U133 Plus 2.0 Array, HG-U133_Plus_2, https://www.ncbi.nlm.nih.gov/geo/query/acc.cgi?acc=GPL570, accessed on 3 July 2021). The detailed information of datasets is as follows: GSE9750 included 33 cervical tumor and 24 normal tissue samples. GSE7803 covered 21 cervical tumor and 10 normal tissue samples. GSE63514 included data from 28 cervical tumor and 24 normal samples. GSE17025 covered 91 endometrial tumor and 12 normal tissue samples. GSE115810 comprised 24 endometrial tumor and 3 normal samples. GSE36389 consisted of 13 endometrial tumor and 7 normal samples.

### 2.2. Data Processing

A robust multi-array average (RMA) approach in the R *affy* package (Version 1.70) [21,22] was performed for background correction and normalization. Then, the normalized cervical and endometrial cancers gene expression data were merged and adjusted by batches by using ComBat function from the R *sva* package (Version 3.40) [23], respectively. For each given microarray platform, GEO provides the annotation details that contain the probe ID and gene id/symbol. Using the annotation table, the probes were easily converted into the corresponding gene symbols. For genes that were mapped by multiple probes, only those with highest average value across all samples were kept. All log2 transformed gene expression values were subjected to z-score standardization.

### 2.3. Differentially Gene Expression Analysis

The DEGs between tumor and normal tissue samples were screened using the R package *limma* (Version 3.48) [24]. The *p* value was adjusted to control the false discovery rate (FDR) based on the method of Benjamini and Hochberg. A false discovery rate (FDR) < 0.05 and |log2 fold change (FC)| > 1 was set as the criteria. Volcano plot and heatmap for DEGs were plotted by performing the R package ggplot2 (https://cran.r-project.org/web/packages/ggplot2/index.html, Version 3.3.5, accessed on 3 July 2021) and *pheatmap* (https://cran.r-project.org/web/packages/pheatmap/index.html, Version 1.0.12, accessed on 3 July 2021). The 78 common DEGs among the two cancers were obtained for further analysis using a Venn diagram.

### 2.4. Gene Ontology (GO) and Kyoto Encyclopedia of Genes and Genomes (KEGG) Enrichment Analysis of DEGs

GO functional annotation and KEGG pathway (Release 98.0) analysis were conducted and visualized by using the R package *clusterProfler* (Version 4.0.5) [25] to understand biological meaning and key pathways associated with the DEGs of gynecologic cancers. The GO functional enrichment analysis of DEGs was divided into three parts: biological process (BP), cellular component (CC), and molecular function (MF). The significant GO terms and KEGG pathways were selected with *p* < 0.05.

### 2.5. Feature Selection Using the LASSO Regression Model

LASSO is a regression analysis method that performs both gene selection and classification. To select candidate cancer-related gene combinations that were reliably associated with gynecologic cancers (cervix and endometrium), the R package *glmnet* (Version 4.1.2) [26] was used to fit a logistic LASSO regression model on the 78 DEGs. In our study, 10-fold cross-validation was performed for tuning parameter selection, and the partial likelihood deviance met the minimum criteria. Using the *ROCR* package (Version 1.0.11) in R, the area under the receiver operating characteristic (ROC) curve was calculated to detect the accuracy of the predictive survival model.

### 2.6. Integration of Protein–Protein Interaction (PPI) Network

The potential relationship among these 20 feature genes was analyzed using Search Tool for the Retrieval of Interacting Genes (STRING, https://string-db.org/, version 11.5, accessed on 3 July 2021), which is a commonly used online tool designed to search for known proteins and evaluate PPI information. The PPI network was then plotted.

### 2.7. Validation of Hub Genes Using Survival Analysis

Survival analysis of hub genes was achieved using the gene expression profiling interactive analysis 2 (GEPIA2) online tool (http://gepia2.cancer-pku.cn/#index, accessed on 3 July 2021), which is based on TCGA datasets. In this study, we utilized the GEPIA2 database to validate the expression of hub genes and explore the relationship between expression value and survival time in 292 CESC patients and 172 UCEC patients. Survival curves were plotted by the expression profiles from TCGA-CESC and -UCEC patients, which were divided into two groups based on the average expression value of each gene (high vs. low). A *p*-value less than 0.05 was considered as statistically significant.

## 3. Results

### 3.1. Identification of the Common DEGs in Cervical and Endometrial Carcinoma

**Figure 1:**
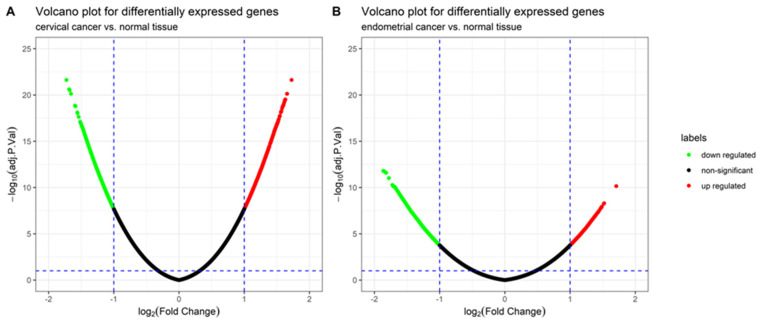
Volcano plot for the differentially expressed genes (DEGs) in two gynecologic tumor types: (**A**) The volcano plot of 920 DEGs between cervical cancer and normal tissue, including the 572 upregulated genes (red color) and 348 downregulated genes (green color). (**B**) The volcano plot of 843 DEGs between endometrial cancer and normal tissue, including the 213 upregulated genes (red color) and 630 downregulated genes (green color).

**Figure 2:**
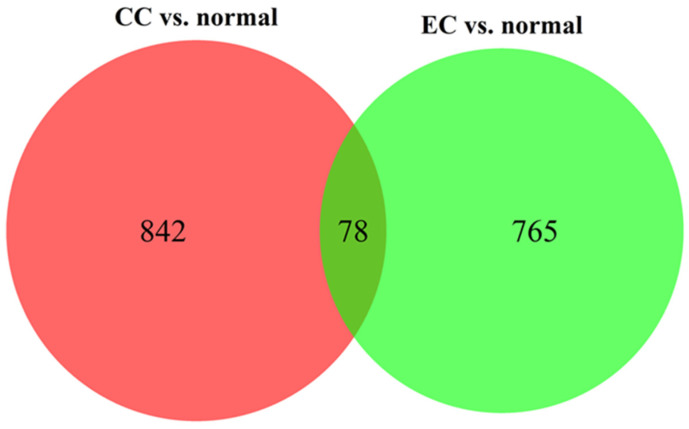
Venn diagram of the relationship between DEGs groups (CC vs. normal, EC vs. normal). The numbers marked in the diagram indicate the number of significant DEGs. CC, cervical cancer; EC, endometrial cancer.

**Figure 3:**
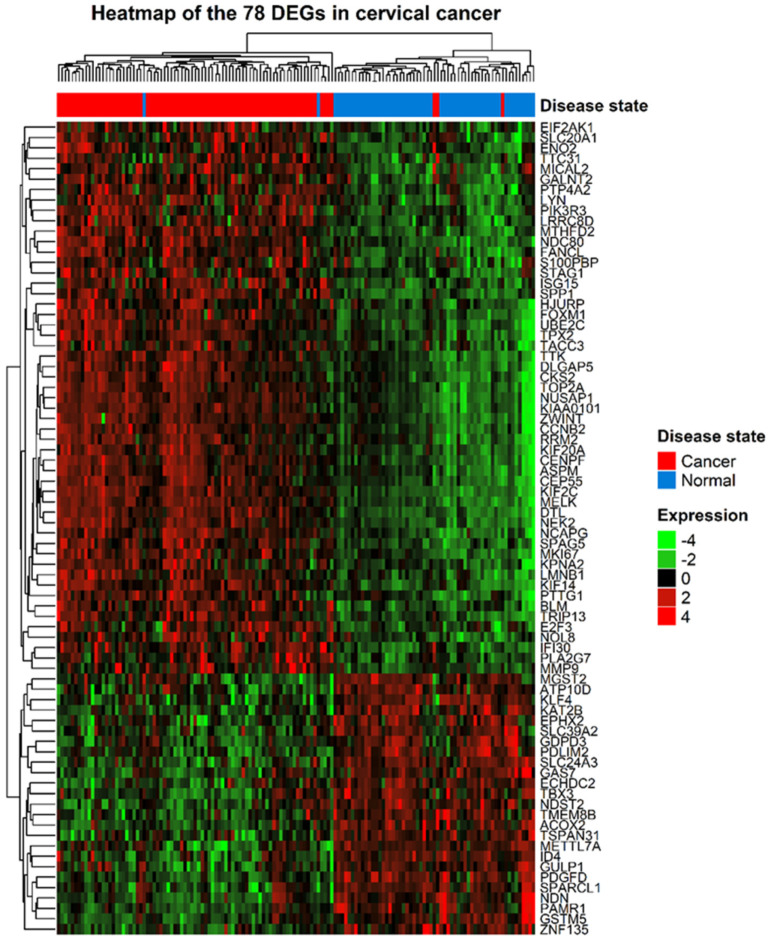
Heatmap of the 78 DEGs between cervical cancer and normal samples. Each row represents a DEG and each column represents a sample. The color represents the raw Z-score ranging from green (low expression) to red (high expression).

**Figure 4:**
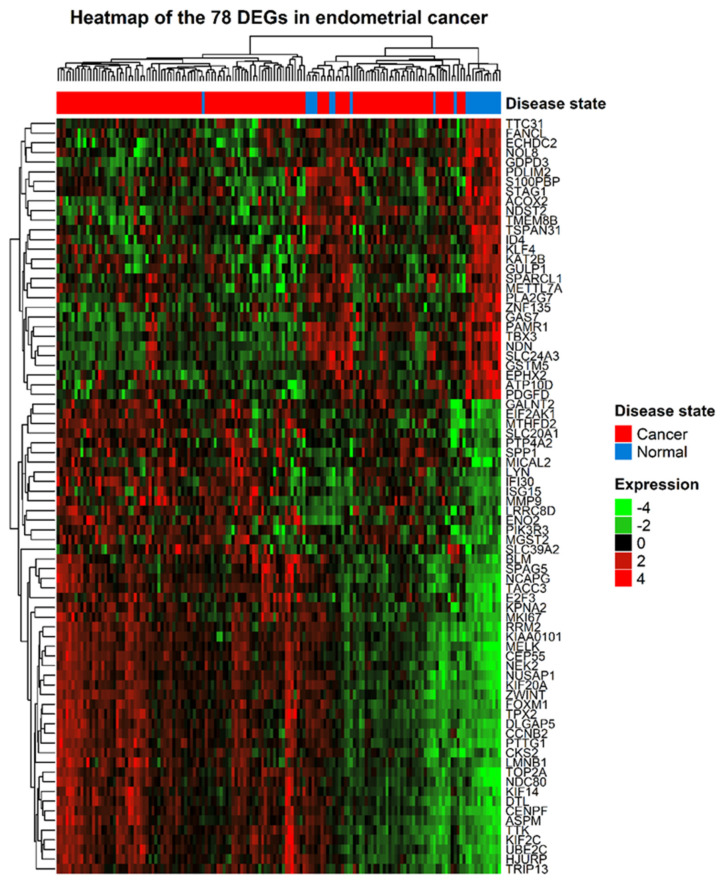
Heatmap of the 78 DEGs between endometrial cancer and normal samples. Each row represents a DEG and each column represents a sample. The color represents the raw Z-score ranging from green (low expression) to red (high expression).

The gene expression profiles and corresponding clinical information for 210 gynecologic cancers and 80 normal tissues were obtained from the GEO database. Using the R package *limma*, 920 (572 upregulated and 348 downregulated) and 843 (213 upregulated and 630 downregulated) DEGs were identified in cervical (Figure 1A) and endometrial (Figure 1B) cancers, respectively. A total of 78 common DEGs among the two gynecologic tumor types are shown in Figure 2, and the expression profiles of 78 DEGs in each dataset was visualized by hierarchical clustering analysis. Sample clustering was also performed, with red representing tumor samples and blue representing normal samples annotated at the left of the plot (Figure 3 and Figure 4). Detailed information of these up- and downregulated genes is shown in Supplementary Tables S1 and S2.

### 3.2. GO and KEGG Enrichment Analysis of the Common 78 DEGs

**Figure 5:**
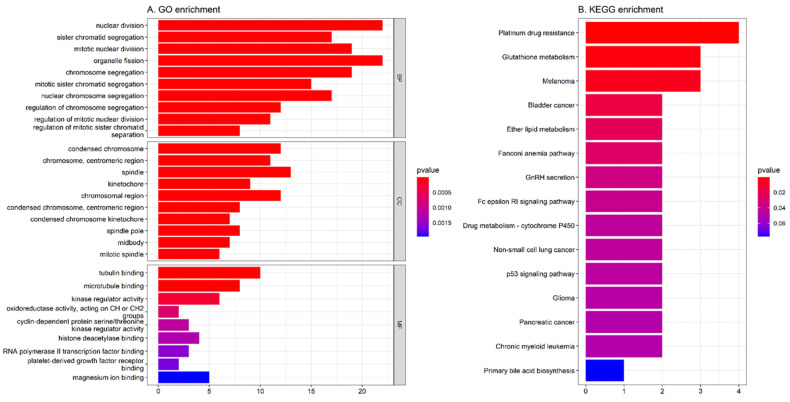
Gene function enrichment analysis: (**A**) GO functional enrichment analysis of 78 DEGs associated with three gynecologic tumor types. (**B**) KEGG enrichment analysis of 78 DEGs associated with three gynecologic tumor types. BP, biological process; CC, cellular component; MF, molecular function.

Gene enrichment analysis was used for the identification of 78 DEGs involved in known pathways and functional association. As demonstrated in Figure 5A, the result of GO functional enrichment analysis shows that DEGs are mostly enriched in nuclear division, organelle fission (BP), spindle (CC), and tubulin binding (MF). The KEGG analysis result reveals that most DEGS were enriched in platinum drug resistance and glutathione metabolism (Figure 5B).

### 3.3. Selection of Significant Genes in Gynecologic Tumor Types Using the LASSO Regression Model

**Figure 6:**
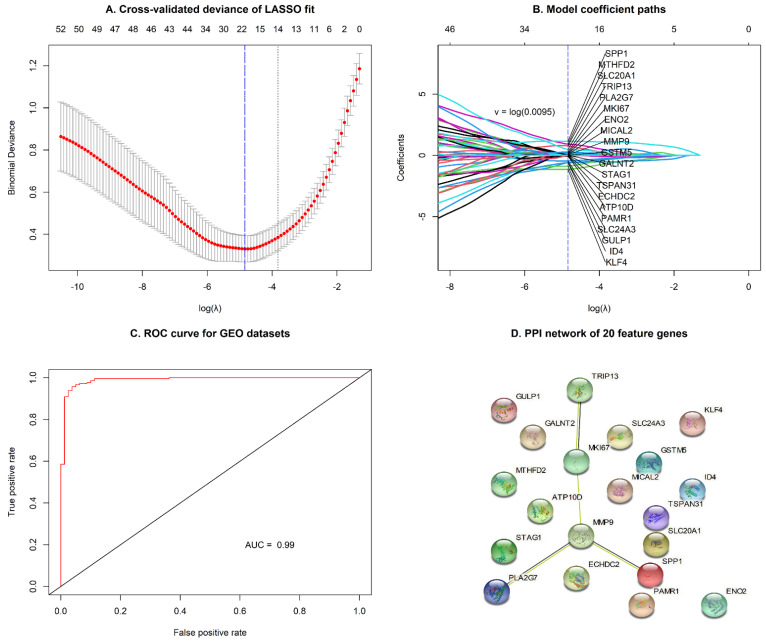
Feature selection using the logistic LASSO regression model by 10-fold cross-validation and the minimum criterion: (**A**) A coefficient profile plot was generated against the log (lambda) sequence. (**B**) LASSO coefficients of 20 significant genes in gynecologic cancers. The left blue vertical line represents the minimum error. (**C**) The receiver operating characteristic (ROC) curve showed the accuracy of the predictive survival model by the area under curve (AUC) for GEO datasets (GSE9750, GSE7803, GSE63514 GSE17025, GSE115810, and GSE36389). (**D**) The PPI network of the 20 feature genes.

A total of 78 DEGs were selected between two groups to fit a LASSO regression model. The next step was to find the most appropriate values for λ (=0.0095) using 10-fold cross-validation (Figure 6A). Finally, 20 genes (SPP1, MTHFD2, SLC20A1, TRIP13, PLA2G7, MKI67, ENO2, MICAL2, MMP9, GSTM5, GALNT2, STAG1, TSPAN31, ECHDC2, ATP10D, PAMR1, SLC24A3, GULP1, ID4, and KLF4) with non-zero coefficients were identified in cervical and endometrial cohorts (Figure 6B; Supplementary Table S3), and used for survival analysis. The accuracy of the predictive survival model based on 20 selected genes was confirmed by the area under curve (AUC = 0.99), as shown in Figure 6C. In addition, the PPI network of the 20 feature genes is shown in Figure 6D.

### 3.4. Verification of Prognostic Value for 20 Significant Genes

**Figure 7:**
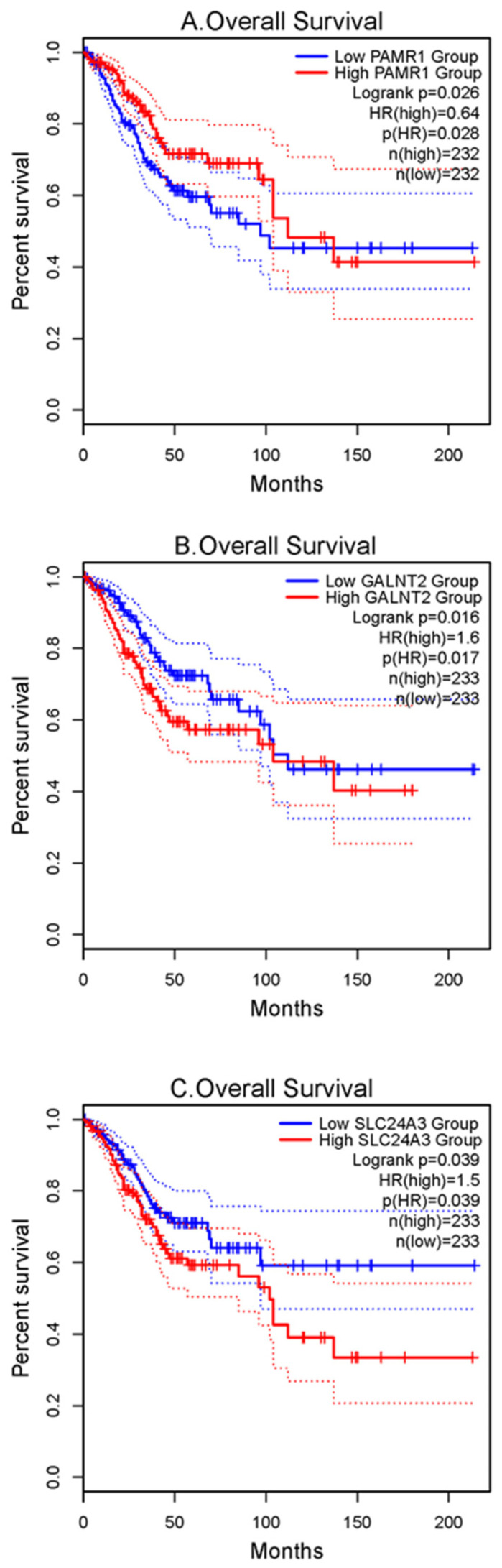
Survival analyses of hub genes were performed using gene expression profiling interactive analysis 2 (GEPIA2) online tool: Survival curves show that (**A**) PAMR1 with high expression level, and (**B**) GALNT2 and (**C**) SLC24A3 with low expression level were significantly associated with better outcomes in patients with TCGA-CESC and -UCEC. CESC, cervical squamous cell carcinoma and endocervical adenocarcinoma; UCEC, uterine corpus endometrial carcinoma.

To verify the prognostic value of the 20 significant genes identified by the LASSO regression model, OS analysis was performed using TCGA-CESC and -UCEC datasets using the GEPIA2 online tool. Among the 20 significant genes, three genes, PAMR1, GALNT2, and SLC24A3, were found to be statistically significant in survival rates between the two groups of patients (*p* < 0.05) (Figure 7; Supplementary Figure S1). Survival curves showed that the higher expression of PAMR1 was associated with better prognosis of patients, as was the lower expression of GALNT2 and SLC24A3.

**Figure 8:**
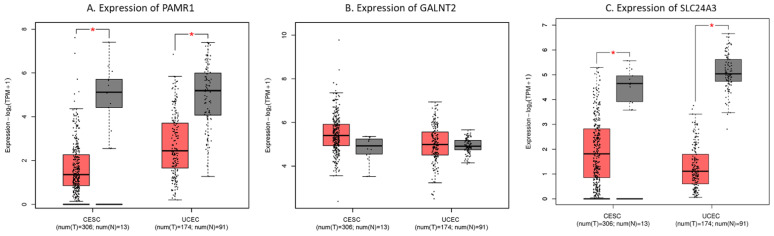
The expression levels of hub genes in TCGA-CESC and -UCEC types using GEPIA2 online tool. Boxplot shows (**A**) PAMR1, (**B**) GALNT2, and (**C**) SLC24A3 expression in tumor (T, red box) and normal tissues (N, grey box), respectively. The asterisk (*) indicates significant difference in comparison with normal samples (*p* < 0.05). CESC, cervical squamous cell carcinoma and endocervical adenocarcinoma; UCEC, uterine corpus endometrial carcinoma. T, tumor; N, normal.

In addition, we compared the mRNA expression of PAMR1, GALNT2, and SLC24A3 in TCGA-CESC and -UCEC types. The boxplot demonstrated that mRNA expression levels of PAMR1 and SLC24A3 were significantly lower in tumor tissues than in normal samples (*p* < 0.05) (Figure 8; Supplementary Figure S2). Finally, the results suggested that PAMR1 and SLC24A3 may act as prognostic biomarkers for gynecologic cancer in our study.

## 4. Discussion

Despite great efforts to study the molecular mechanisms of two types of gynecologic cancer (CC and EC), the precise biomarkers and targeted therapy for patients with CC and EC remain limited. To investigate potential biomarkers for better detection and therapy, we integrated the gene expression profiles of GSE9750, GSE7803, GSE63514, GSE17025, GSE115810, and GSE36389, which contained 210 tumor samples from cervix and endometrium and 80 normal tissues.

In this study, 78 DEGs were identified with the criteria of FDR < 0.05 and |log_2_ FC| > 1 in both cancer types. As a result of the functional annotation obtained by the R package *clusterProfiler*, the GO enrichment analysis revealed that the 78 DEGs were mainly associated with “nuclear division”, “condensed chromosome”, and “tubulin binding”. The KEGG pathway analysis showed “platinum drug resistance” and “glutathione metabolism” pathways were significantly enriched, which suggests that these genes may be involved in the action process or metabolic reaction of drugs. Notably, multiple different cancer-association pathways, including “melanoma”, “bladder cancer”, and “non-small cell lung cancer”, were also identified. This indicates gynecologic cancer types may exhibit similar molecular mechanisms with tumor types of other systems. With the LASSO logistic regression model, 20 hub genes (SPP1, MTHFD2, SLC20A1, TRIP13, PLA2G7, MKI67, ENO2, MICAL2, MMP9, GSTM5, GALNT2, STAG1, TSPAN31, ECHDC2, ATP10D, PAMR1, SLC24A3, GULP1, ID4, and KLF4) were identified and could effectively distinguish between gynecologic cancer and normal tissues. Similar to previous studies, SPP1 [27,28], MTHFD2 [29,30], TRIP13 [31,32], MKI67 [33,34], MMP9 [35,36], and KLF4 [37,38] were some of the most clinically valuable tumor markers, especially in CC and EC.

Moreover, using GEPIA2, the expression of PAMR1 and SLC24A3 in tumor tissues was downregulated significantly compared with the normal tissue and was related to the OS of patients with gynecologic cancer. The results demonstrated that PAMR1 and SLC24A3 may serve as potential prognostic biomarkers in gynecologic cancer.

Peptidase domain containing associated with muscle regeneration 1 (PAMR1) is a tumor suppressor gene and expressed in various tissues such as skeletal muscle [39,40], brain [40], and mammary gland [39,41]. Many studies have proven that overexpression of PAMR1 markedly suppresses cell growth, especially in breast cancer [39,41], polycystic ovary syndrome [42], EC [43], and CC [40]. Consistent with our observation, the suppression of PAMR1 can lead to poor prognosis and an increased risk of death.

Solute carrier family 24 member 3 (SLC24A3), also known as NCKX3, is a sodium–calcium regulator of cells [44,45]. Its expression is most abundant within the human endometrium at the mRNA and protein levels, and plays a role in the reproductive function of the endometrium [46,47]. In accordance with our results, high SLC24A3 levels are associated with poor prognosis. Thus, angiogenesis has an important impact on the pathogenesis of gynecologic cancer.

## 5. Conclusions

In our research, 78 DEGs were identified in gynecologic cancer. The LASSO regression model and survival analysis further suggested that two hub genes (PAMR1 and SLC24A3) could serve as potential biomarkers for the treatment or diagnosis of cervical and endometrial cancers. More work is need to clarify the pathogenesis of gynecologic cancer in which these genes are involved and to validate their value as prognostic biomarkers both in vitro and in vivo.

Furthermore, limitations of our study include the lack of analysis of the clinical characteristics of gynecologic cancer, such as grade, stage, and lymph node metastasis. In future investigations, we will further explore hub genes and their potential function based on detailed clinical information.

## Figures and Tables

**Table 1:** The detail of gene expression profiles of cervical cancer (CC) (GSE9750, GSE7803, GSE63514) and endometrial cancer (EC) (GSE17025, GSE115810, GSE36389).

Datasets	Tissues	Tumor	Normal	Platform
GSE9750	cervix	33	24	GPL96
GSE7803		21	10	GPL96
GSE63514		28	24	GPL570
GSE17025	endometrium	91	12	GPL570
GSE115810		24	3	GPL96
GSE36389		13	7	GPL96

## Data Availability

The datasets used and analyzed in this study were available from Gene Expression Omnibus (GEO, https://www.ncbi.nlm.nih.gov/geo/ accessed on 3 July 2021) and The Cancer Genome Atlas (TCGA, https://portal.gdc.cancer.gov/ accessed on 3 July 2021).

## Supplementary Materials

The following are available online at https://www.mdpi.com/article/10.3390/jpm11111177/s1, Figure S1: Survival analyses of 17 genes were performed using gene expression profiling interactive analysis 2 (GEPIA2) online tool. (A) ATP10D (B) ECHDC2 (C) ENO2 (D) GSTM5 (E) GULP1 (F) ID4 (G) KLF4 (H) MICAL2 (I) MKI67 (J) MMP9 (K) MTHFD2 (L) PLA2G7 (M) SLC20A1 (N) SPP1 (O) STAG1 (P) TRIP13 (Q) TSPAN31, Figure S2: The expression of 17 genes in TCGA-CESC and -UCEC types were performed using gene expression profiling interactive analysis 2 (GEPIA2) online tool. (A) ATP10D (B) ECHDC2 (C) ENO2 (D) GSTM5 (E) GULP1 (F) ID4 (G) KLF4 (H) MICAL2 (I) MKI67 (J) MMP9 (K) MTHFD2 (L) PLA2G7 (M) SLC20A1 (N) SPP1 (O) STAG1 (P) TRIP13 (Q) TSPAN31. The asterisk (*) indicates significant difference in comparison with normal samples (*p* < 0.05), Table S1: The DEGs between cervical cancers and normal tissues were identified by Limma, Table S2: The DEGs between endometrial cancers and normal tissues were identified by Limma, Table S3: Lasso regression coefficients values for 78 common genes.
