# Supplementary material for: LASSO and Bioinformatics Analysis in the Identification of Key Genes for Prognostic Genes of Gynecologic Cancer
Source: J Pers Med. 2021 Nov 11;11(11):1177. doi: 10.3390/jpm11111177 (PMC8617991; doi:10.3390/jpm11111177)
Supplement: Supplementary file 1 [file jpm-11-01177-s001.zip › Supplementary Figure 1.pdf]

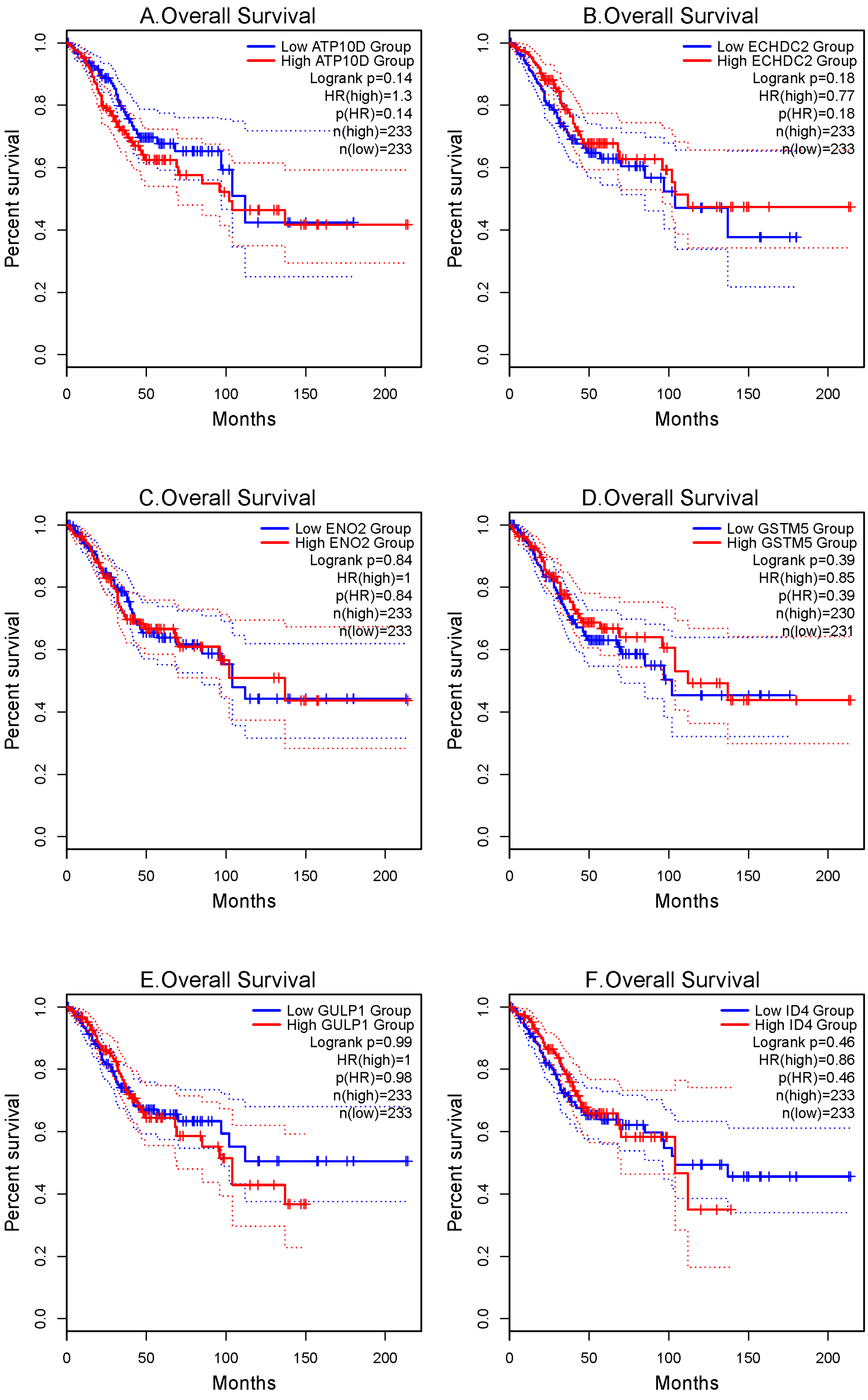
A.Overall Survival
Low ATP10D Group
High ATP10D Group
Logrank p=0.14
HR(high)=1.3
p(HR)=0.14
n(high)=233
n(low)=233
B.Overall Survival
Low ECHDC2 Group
High ECHDC2 Group
Logrank p=0.18
HR(high)=0.77
p(HR)=0.18
n(high)=233
n(low)=233
C.Overall Survival
Low ENO2 Group
High ENO2 Group
Logrank p=0.84
HR(high)=1
p(HR)=0.84
n(high)=233
n(low)=233
D.Overall Survival
Low GSTM5 Group
High GSTM5 Group
Logrank p=0.39
HR(high)=0.85
p(HR)=0.39
n(high)=230
n(low)=231
E.Overall Survival
Low GULP1 Group
High GULP1 Group
Logrank p=0.99
HR(high)=1
p(HR)=0.98
n(high)=233
n(low)=233
F.Overall Survival
Low ID4 Group
High ID4 Group
Logrank p=0.46
HR(high)=0.86
p(HR)=0.46
n(high)=233
n(low)=233
Percent survival
0.0
0.2
0.4
0.6
0.8
1.0
Months
0
50
100
150
200

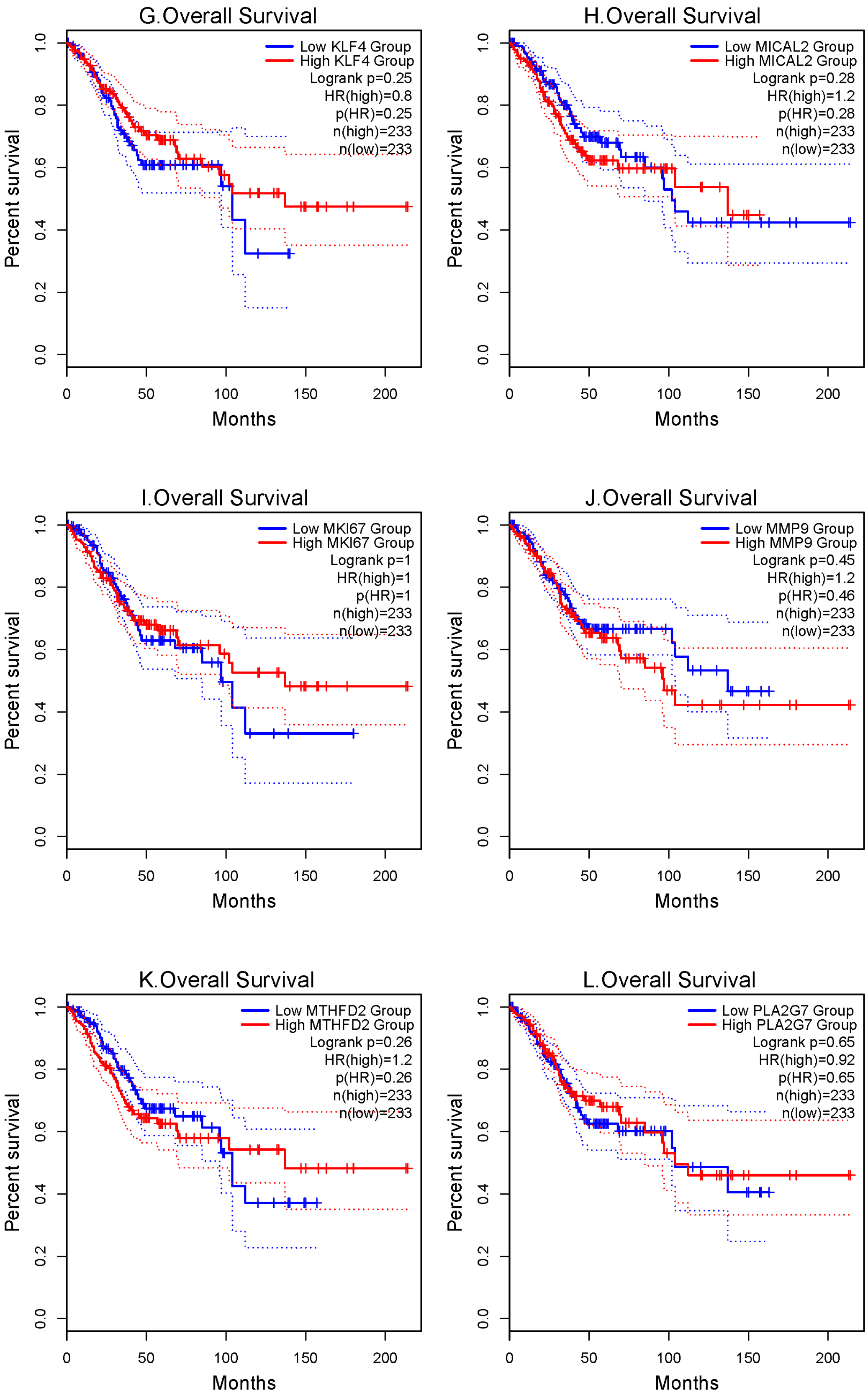
G.Overall Survival
Low KLF4 Group
High KLF4 Group
Logrank p=0.25
HR(high)=0.8
p(HR)=0.25
n(high)=233
n(low)=233
H.Overall Survival
Low MICAL2 Group
High MICAL2 Group
Logrank p=0.28
HR(high)=1.2
p(HR)=0.28
n(high)=233
n(low)=233
I.Overall Survival
Low MKI67 Group
High MKI67 Group
Logrank p=1
HR(high)=1
p(HR)=1
n(high)=233
n(low)=233
J.Overall Survival
Low MMP9 Group
High MMP9 Group
Logrank p=0.45
HR(high)=1.2
p(HR)=0.46
n(high)=233
n(low)=233
K.Overall Survival
Low MTHFD2 Group
High MTHFD2 Group
Logrank p=0.26
HR(high)=1.2
p(HR)=0.26
n(high)=233
n(low)=233
L.Overall Survival
Low PLA2G7 Group
High PLA2G7 Group
Logrank p=0.65
HR(high)=0.92
p(HR)=0.65
n(high)=233
n(low)=233
Percent survival
Months
0.0
0.2
0.4
0.6
0.8
1.0
0
50
100
150
200

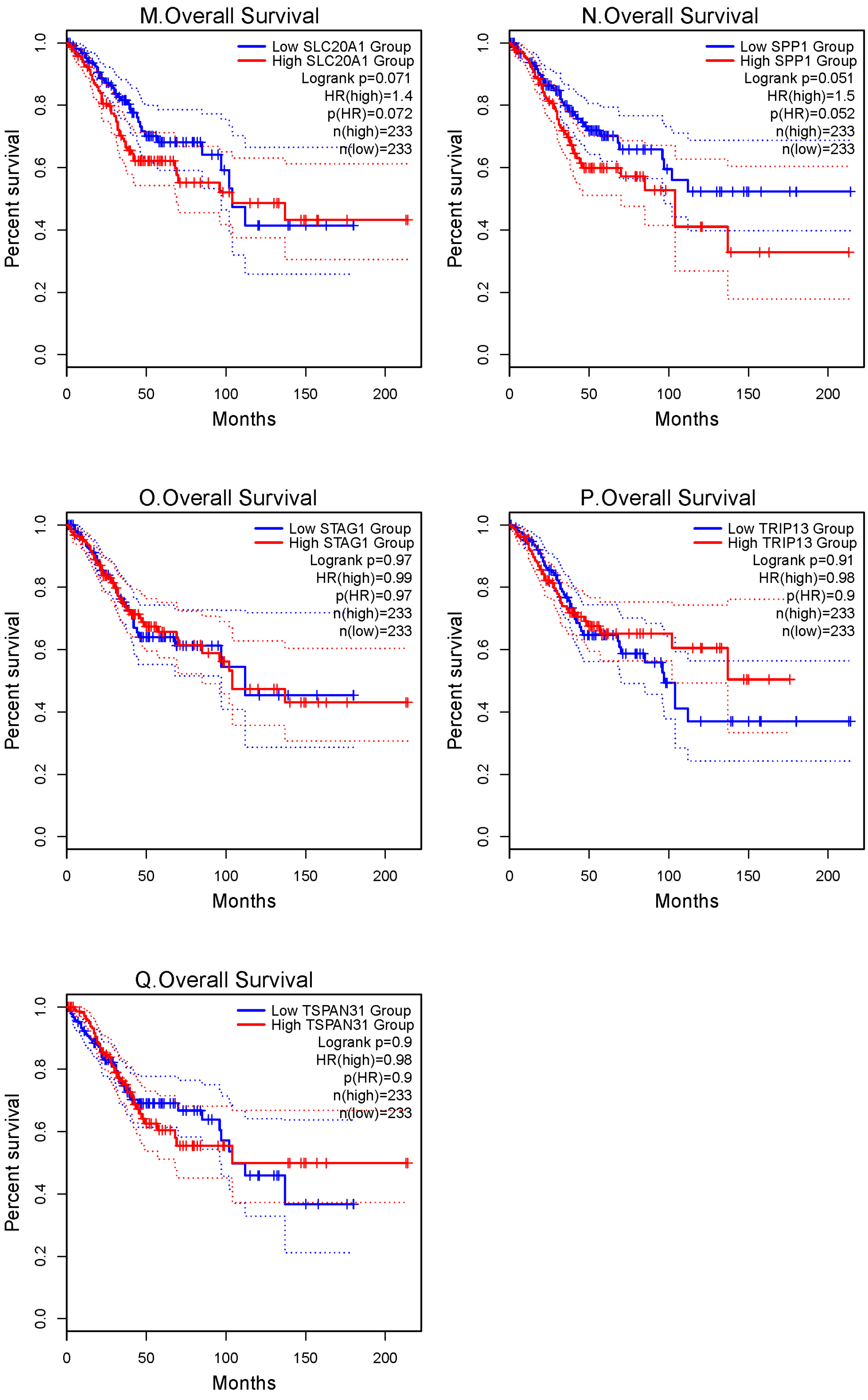

Supplementary Figure S1. Survival analyses of 17 genes were performed using gene expression profiling interactive analysis 2 (GEPIA2) online tool. (A) ATP10D (B) ECHDC2 (C) ENO2 (D) GSTM5 (E) GULP1 (F) ID4 (G) KLF4 (H) MICAL2 (I) MKI67 (J) MMP9 (K) MTHFD2 (L) PLA2G7 (M) SLC20A1 (N) SPP1 (O) STAG1 (P) TRIP13 (Q) TSPAN31.
