# Supplementary material for: LASSO and Bioinformatics Analysis in the Identification of Key Genes for Prognostic Genes of Gynecologic Cancer
Source: J Pers Med. 2021 Nov 11;11(11):1177. doi: 10.3390/jpm11111177 (PMC8617991; doi:10.3390/jpm11111177)
Supplement: Supplementary file 1 [file jpm-11-01177-s001.zip › Supplementary Figure 2-CN.pdf]

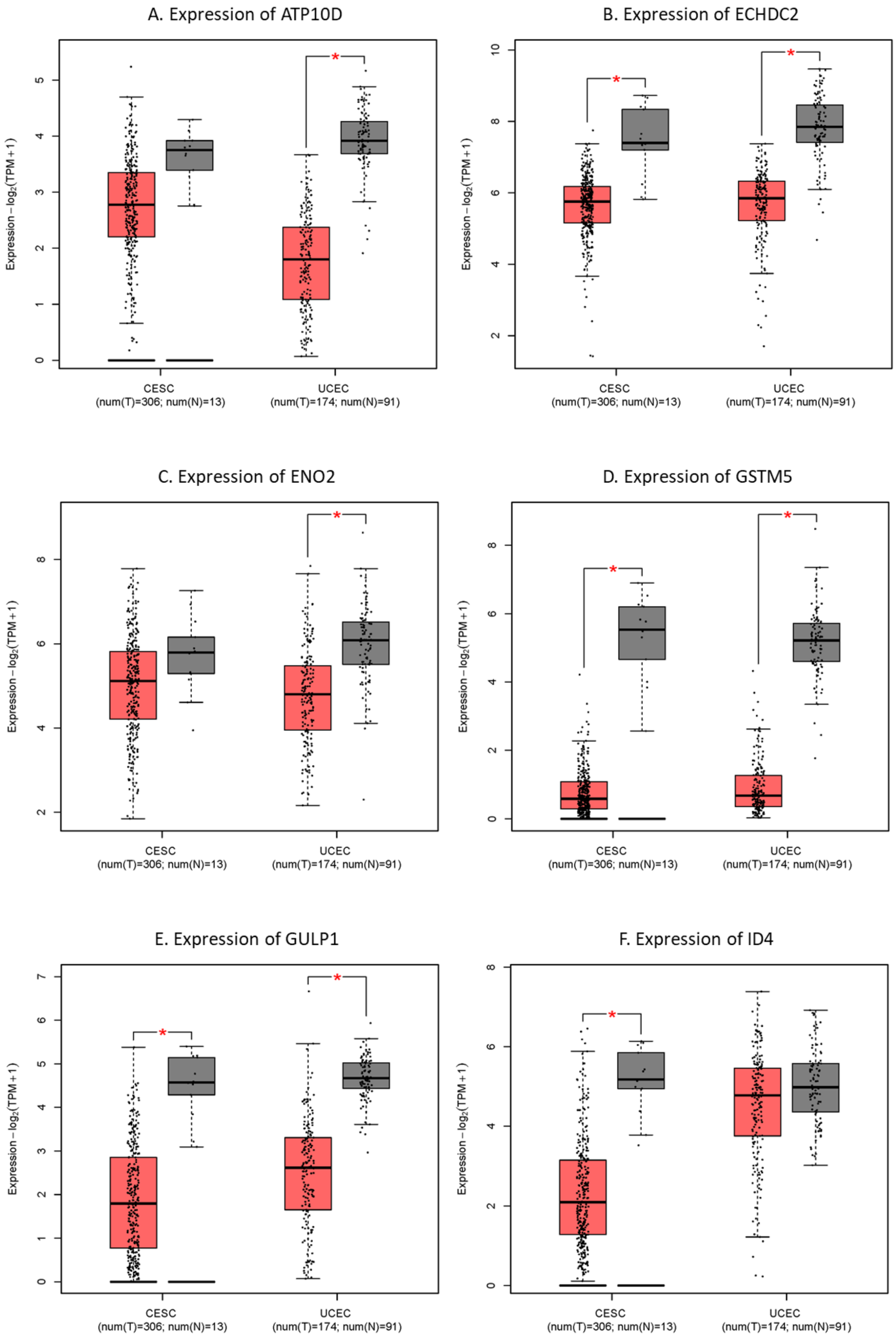
A. Expression of ATP10D
B. Expression of ECHDC2
C. Expression of ENO2
D. Expression of GSTM5
E. Expression of GULP1
F. Expression of ID4
Expression – log2(TPM + 1)
CESC
(num(T)=306; num(N)=13)
UCEC
(num(T)=174; num(N)=91)
*

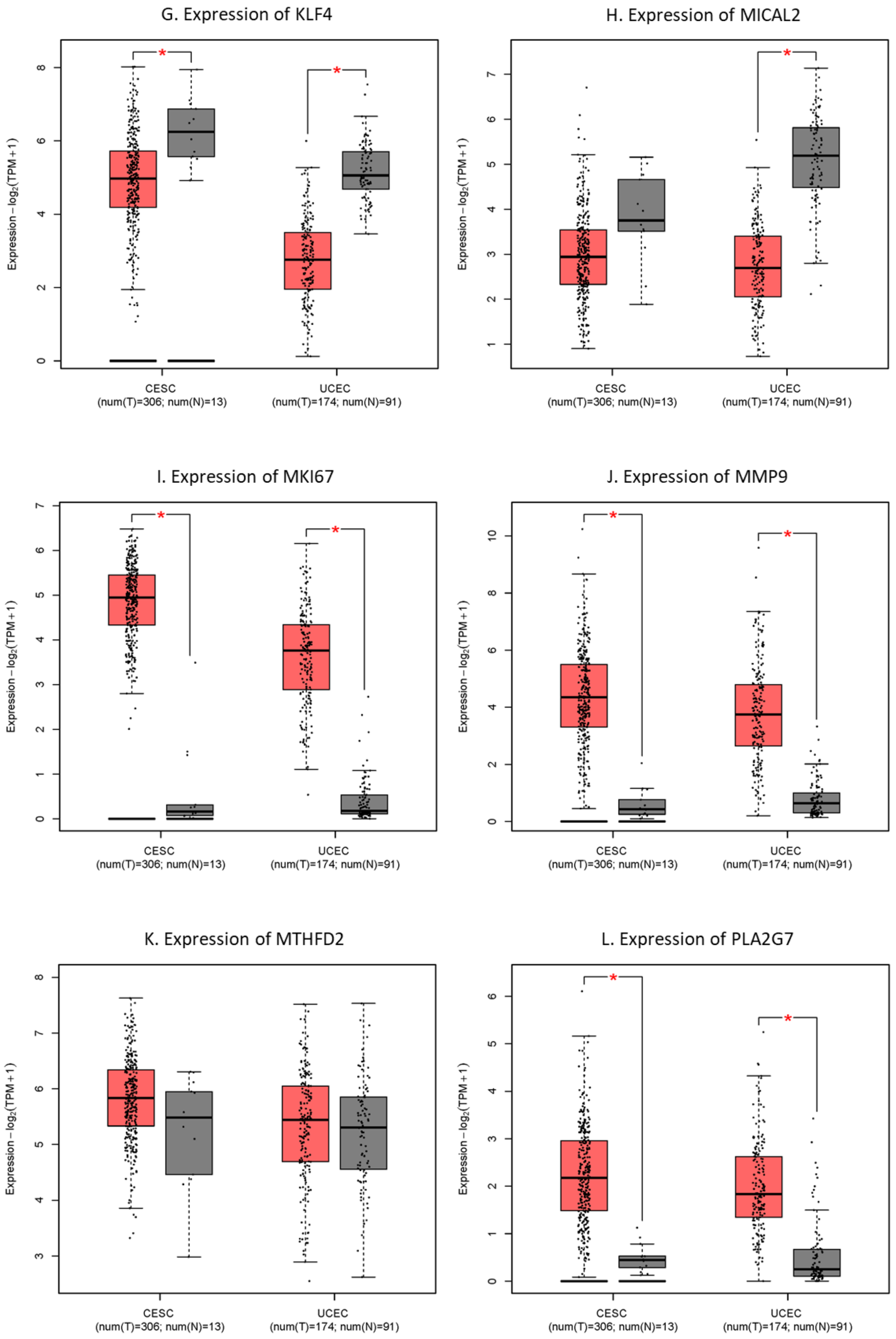
G. Expression of KLF4
H. Expression of MICAL2
I. Expression of MKI67
J. Expression of MMP9
K. Expression of MTHFD2
L. Expression of PLA2G7
Expression − log2(TPM + 1)
*
CESC
(num(T)=306; num(N)=13)
UCEC
(num(T)=174; num(N)=91)

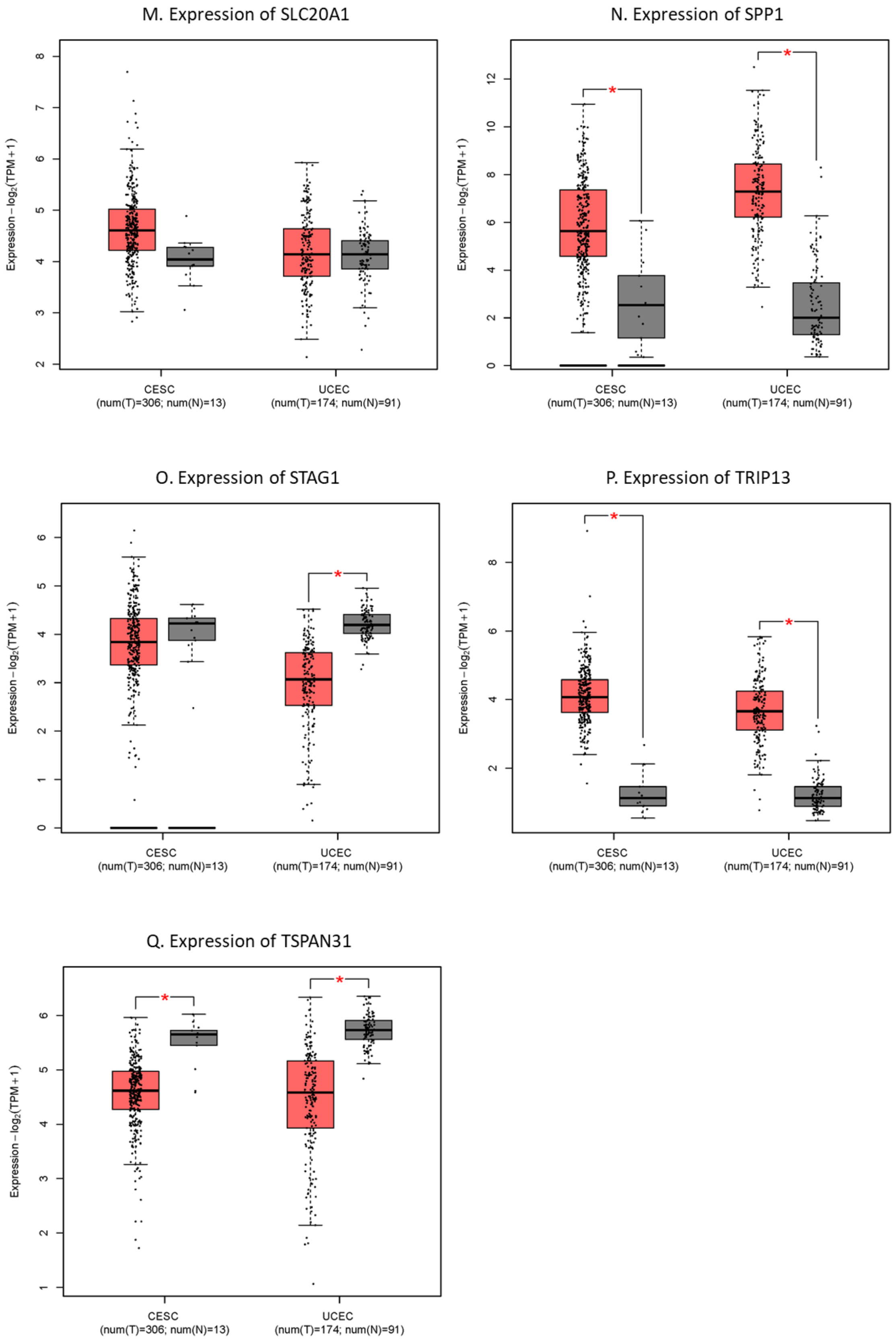

Supplementary Figure S2. The expression of 17 genes in TCGA-CESC and -UCEC types were performed using gene expression profiling interactive analysis 2 (GEPIA2) online tool. (A) ATP10D (B) ECHDC2 (C) ENO2 (D) GSTM5 (E) GULP1 (F) ID4 (G) KLF4 (H) MICAL2 (I) MKI67 (J) MMP9 (K) MTHFD2 (L) PLA2G7 (M) SLC20A1 (N) SPP1 (O) STAG1 (P) TRIP13 (Q) TSPAN31. The asterisk (*) indicates significant difference in comparison with normal

samples ($p < 0.05$).
